# SOD2 deficiency promotes aging phenotypes in mouse skin

**DOI:** 10.18632/aging.100433

**Published:** 2012-02-11

**Authors:** Urbain Weyemi, Palak R. Parekh, Christophe E. Redon, William M. Bonner

**Affiliations:** Laboratory of Molecular Pharmacology, CCR, NCI, NIH, Bethesda MD, USA

**Keywords:** SOD2, free radicals, senescence, skin aging

Reactive oxygen species (ROS) are essential for survival but also pose serious risks to that survival. A particularly striking example was the demonstration in 2003 by the Campisi group that primary mouse fibroblasts have an indefinite proliferative lifespan in 3% oxygen, the amount found in the capillaries feeding the tissues, but greatly shortened ones under normal in vitro culturing conditions, i. e., 20% oxygen [1]. Now, the same group has generated some insights into how oxidative stress contributes to cellular senescence and aging phenotypes in mouse skin [2].

ROS may be produced in a regulated manner during cellular metabolism but they can also arise in an unregulated manner by metabolic dysfunctions and by exogenous stresses. ROS production is typically localized in cellular compartments by NAD(P)H oxidases, lipoxygenases, cyclooxygenases among others as well as by the mitochondrial electron-transport chain [3]. At physiological levels, ROS play beneficial or even essential roles as regulatory mediators in signalling or defence processes, including the promotion of endothelium-dependent vasorelaxation, apoptosis, angiogenesis, erythropoietin production and destruction of bacteria and other foreign substances by macrophages [4].

However, compromised homeostatic pathways lead to elevated ROS levels that may result in the damage of cellular components (i.e., proteins, lipids, DNA). A growing body of evidence implicates oxidative stress in both aging and a wide spectrum of human diseases including diabetes, atherosclerosis, hypertension, cancer, cardiovascular diseases and neurodegenerative diseases among others [5].

The counterpoint of deleterious and useful ROS roles may have provided evolutionary pressure to develop robust pathways for intracellular redox homeostasis including antioxidants such as glutathione peroxidases, peroxiredoxins, catalases and SODs. The first line of defence against ROS appears to be one of the superoxide dismutases, SOD2, (aka manganese superoxide dismutase (MnSOD)). SOD2 is a mitochondrial matrix protein that converts the superoxide anion (O_2_^−^) to hydrogen peroxide (H_2_O_2_), which is in turn, converted to molecular oxygen and water by catalase and peroxiredoxins. In this Aging paper, the Campisi group showed that normal aged mice exhibit impaired mitochondrial complex II activity and increased frequencies of senescent cells. They observed similar phenotypes in SOD2 knockout (KO) mice at very young ages and also reported that these mice exhibit significant epidermal thinning which is an age-associated phenotype in humans as well as mice. The epidermal thinning appeared to be due to decreased cellular proliferation in the senescent skin tissue rather than increased apoptosis. In addition, senescence in SOD2 KO mouse skin correlated with increased levels of two senescence markers, SA-βgal activity and the p16^INK4a^ tumor suppressor, as well as amplified keratinocyte terminal differentiation (Figure 1). Moreover, these features are accompanied by the presence of nuclear DNA damage in the epidermis, a characteristic linked to senescence in both mouse and human cells in culture [6].

**Figure 1:**
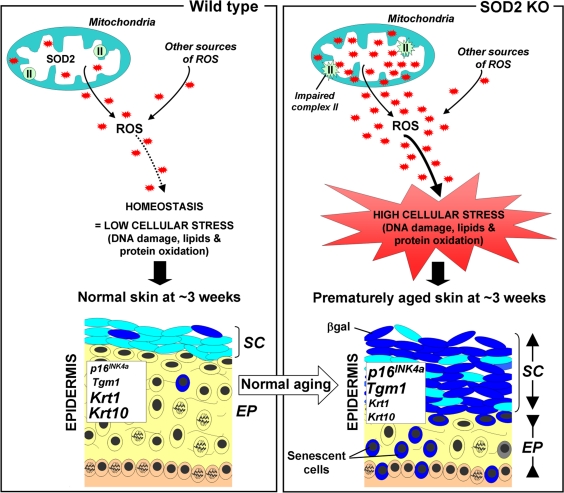
Scheme illustrating the putative role of SOD2 in skin aging (**Left panel**) In normal mice, SOD2 scavenges superoxide anions O_2_^−^, a byproduct of mitochondrial respiration, resulting in normal levels of reactive oxygen species (ROS, red stars). This homeostasis leads to minimal cellular stress (little damage to DNA, lipids and proteins), resulting in normal skin, characterized by a thin stratum corneum (**SC**) and a thick epidermis (**EP**). (**Right panel**) In contrast, SOD2 deficiency leads to elevated levels of ROS and oxidative stress (increased damage to DNA, proteins and lipids) as well as impaired mitochondrial (complex II) activity. This results in premature aging, characterized by increased thickness of the stratum corneum (**SC**) and thinning of the epidermis (**EP**). The change of the stratum corneum is accompanied by the accumulation of differentiated keratinocytes with high levels of β-galactosidase activity, while the thinning epidermis results from a combination of decreased proliferation and increased senescence in the suprabasal layer (**yellow**). Compared to normal mice, SOD2 KO mice exhibit elevated skin transcript levels of Tgm1 and p16INK4a, markers for differentiated keratinocytes and cellular senescence respectively, as well as decreased transcript levels for keratin 1(krt1) and keratin 10 (krt10), two markers of transit amplifying cells and early differentiating cells.

Similar to the observations made in mice, other studies in yeast and Drosophila also show a predominant role for SOD2 in increasing the lifespan [7-9]. However, the evidence of a direct link between aging and elevated ROS levels is not undisputed. For example, mice heterozygous for SOD2 exhibited accrued DNA damage and subsequent predisposition to cancer, but without any impact on the lifespan [10]. In mice with a mutated mitochondrial DNA polymerase, PolG, ROS production was not significantly increased but the mitochondria accumulated mutations which, in turn, resulted in dysfunctions leading to several aging phenotypes and early death [11, 12].

Other studies failed to find a direct link between SOD2 and aging. A study with mice showed that SOD2 overexpression, while resulting in decreased lipid peroxidation, did not alter either lifespan or age-related pathology [13]. In the nematode, depletion of SOD2 increased oxidative stress but lifespan could still be dramatically prolonged [14]. Also, in the human colorectal cancer cell line HCT116, SOD2 overexpression unexpectedly induced growth arrest and increased senescence [15]. These discrepancies may be explained by the differential responses between species and cell types to a rise in ROS levels.

The shorter lifespan observed in SOD2 KO mice compared to wild-type and heterozygous mice may be reminiscent of previous studies showing that rates of mitochondrial O_2_^−^ and H_2_O_2_ generation were inversely correlated to maximum life span potential with shorter-lived species producing relatively higher amounts of ROS [16].

Because SOD2 plays a role in aging in mice, it would be interesting to look if a correlation exists between age-related pathologies and SOD2 alterations in human. For example, mitochondrial dysfunction was shown to contribute to the pathogenesis of aging-related neurodegenerative diseases [17]. Moreover, presently unresolved issues include the exact nature of oxidant species involved in the establishment of senescence, and the molecular pathways which link mitochondrial oxidative stress to the appearance of genomic DNA lesions. Further research will clarify how these lesions occur in the nucleus, their relationship to aging and which capabilities of cellular redox homeostasis are most important in minimizing them.

## References

[R1] Parrinello S, Samper E, Krtolica A, Goldstein J, Melov S, Campisi J (2003). Oxygen sensitivity severely limits the replicative lifespan of murine fibroblasts. Nat Cell Biol.

[R2] Velarde MC, Flynn JM, Day NU, Melov S, Campisi J (2012). Mitochondrial oxidative stress caused by Sod2 deficiency promotes cellular senescence and aging phenotypes in the skin. Aging (Albany NY).

[R3] Finkel T (2011). Signal transduction by reactive oxygen species. J Cell Biol.

[R4] Valko M, Leibfritz D, Moncol J, Cronin MT, Mazur M, Telser J (2007). Free radicals and antioxidants in normal physiological functions and human disease. Int J Biochem Cell Biol.

[R5] Sedelnikova OA, Redon CE, Dickey JS, Nakamura AJ, Georgakilas AG, Bonner WM (2010). Role of oxidatively induced DNA lesions in human pathogenesis. Mutat Res.

[R6] Nakamura AJ, Chiang YJ, Hathcock KS, Horikawa I, Sedelnikova OA, Hodes RJ, Bonner WM (2008). Both telomeric and non-telomeric DNA damage are determinants of mammalian cellular senescence. Epigenetics Chromatin.

[R7] Sun J, Folk D, Bradley TJ, Tower J (2002). Induced overexpression of mitochondrial Mn-superoxide dismutase extends the life span of adult Drosophila melanogaster. Genetics.

[R8] Bonawitz ND, Rodeheffer MS, Shadel GS (2006). Defective mitochondrial gene expression results in reactive oxygen species-mediated inhibition of respiration and reduction of yeast life span. Mol Cell Biol.

[R9] Unlu ES, Koc A (2007). Effects of deleting mitochondrial antioxidant genes on life span. Ann N Y Acad Sci.

[R10] Van Remmen H, Ikeno Y, Hamilton M, Pahlavani M, Wolf N, Thorpe SR, Alderson NL, Baynes JW, Epstein CJ, Huang TT, Nelson J, Strong R, Richardson A (2003). Life-long reduction in MnSOD activity results in increased DNA damage and higher incidence of cancer but does not accelerate aging. Physiol Genomics.

[R11] Trifunovic A, Hansson A, Wredenberg A, Rovio AT, Dufour E, Khvorostov I, Spelbrink JN, Wibom R, Jacobs HT, Larsson NG (2005). Somatic mtDNA mutations cause aging phenotypes without affecting reactive oxygen species production. Proc Natl Acad Sci U S A.

[R12] Larsson NG (2010). Somatic mitochondrial DNA mutations in mammalian aging. Annu Rev Biochem.

[R13] Jang YC, Perez VI, Song W, Lustgarten MS, Salmon AB, Mele J, Qi W, Liu Y, Liang H, Chaudhuri A, Ikeno Y, Epstein CJ, Van Remmen H, Richardson A (2009). Overexpression of Mn superoxide dismutase does not increase life span in mice. J Gerontol A Biol Sci Med Sci.

[R14] Van Raamsdonk JM, Hekimi S (2009). Deletion of the mitochondrial superoxide dismutase sod-2 extends lifespan in Caenorhabditis elegans. PLoS Genet.

[R15] Behrend L, Mohr A, Dick T, Zwacka RM (2005). Manganese superoxide dismutase induces p53-dependent senescence in colorectal cancer cells. Mol Cell Biol.

[R16] Ku HH, Brunk UT, Sohal RS (1993). Relationship between mitochondrial superoxide and hydrogen peroxide production and longevity of mammalian species. Free Radic Biol Med.

[R17] Filosto M, Scarpelli M, Cotelli MS, Vielmi V, Todeschini A, Gregorelli V, Tonin P, Tomelleri G, Padovani A (2011). The role of mitochondria in neurodegenerative diseases. J Neurol.

